# Genetic alterations in *SUPT6H* are associated with neurodevelopmental disorders

**DOI:** 10.1016/j.bbadis.2026.168226

**Published:** 2026-03-19

**Authors:** Bruno Carabelli, Hyung-Goo Kim, Bonsu Ku, Clara Berdasco, Yu Young Jeong, Tho Lai, Mi-Hyeon Jang, Detlev Boison, Yong Kim

**Affiliations:** a Department of Neurosurgery, Robert Wood Johnson Medical School, Rutgers University, Piscataway, NJ, 08854, United States; b Orphan Disease Therapeutic Target Research Center, Korea Research Institute of Bioscience and Biotechnology, Daejeon, 34141, Republic of Korea; c Brain Health Institute, Rutgers University, Piscataway, NJ, 08854, United States; d Rutgers Cancer Institute, New Brunswick, NJ, 08904, United States

**Keywords:** SUPT6H, *De novo* variants, Neurodevelopmental disorders, Parvalbumin interneurons, Motor defects, Depression-like behavior

## Abstract

Genetic variants affecting the RNA polymerase II complex have been associated with various neuro-developmental disorders (NDDs). SUPT6H, an RNA polymerase II-associated elongation factor and a histone chaperone, plays a critical role in transcriptional regulation. However, the contribution of *SUPT6H* variants to human NDDs and the phenotypic consequences of its loss-of-function *in vivo* remain unexplored. Here, we analyzed 18 published sporadic single-nucleotide variants (SNVs) of *SUPT6H* associated with human developmental disorders. Molecular modeling suggests that these variants are likely deleterious, leading to loss of function. Consistent with this, homozygous or heterozygous *Supt6* null mice exhibit embryonic lethality, underscoring its essential role during development. To investigate the postnatal consequences of Supt6 deficiency, we generated conditional *Supt6* knockout (KO) mice with targeted deletion in parvalbumin-expressing GABAergic interneurons (cKO^PV^). Homozygous *Supt6* cKO^PV^ mice displayed motor defects and behavioral seizures, whereas heterozygous counterparts exhibited behavioral phenotypes relevant to neuropsychiatric disorders despite normal motor activity. Notably, both heterozygous and homozygous *Supt6* cKO^PV^ mice showed a significant reduction in parvalbumin-expressing neurons compared to wild-type controls. These findings establish a direct link between Supt6 loss-of-function and neurodevelopmental phenotypes, highlighting its critical role in maintaining interneuron populations and neural circuit integrity. Altogether, our results suggest that deleterious *SUPT6H* variants may contribute to the etiology of NDDs, providing valuable insights into its function and potential as a therapeutic target.

## 1. Introduction

Genetic mutations affecting fundamental cellular activities are often associated with neurodevelopmental disorders (NDDs) [1]. RNA polymerase II comprises twelve subunits. It is responsible for the transcription of all protein-encoding genes and several long and short non-coding RNAs [2]. Alterations in the function of RNA polymerase II and regulatory proteins, in the processes of initiation, elongation, or termination of gene transcription, have been identified as critical risk factors for NDDs [3,4]. Identifying gene mutations in the transcription units is crucial for understanding the underlying disease mechanisms and finding therapeutic targets for NDDs.

SUPT6H (histone chaperone and transcription elongation factor, also called SPT6) is a critical component in gene transcription as an elongation factor [5–7] and a histone chaperone [7,8]. SUPT6H plays an essential role in multiple stages of the transcription cycle. It ensures the stability of RNA polymerase II during elongation [5–7] and maintains chromatin integrity [5] by influencing nucleosome assembly [5] and histone modifications [8,9]. By interacting with the H3K36 histone methyltransferase SETD2 (SET Domain Containing 2), together with IWS1 (Interacts With SUPT6H) [6,7,9], SUPT6H regulates histone marks that are critical for chromatin organization and transcriptional fidelity [9]. Notably, mutations in *SETD2* are known to cause several NDDs, including autism spectrum disorder (ASD) [10–12], intellectual disability [12–14], Dravet syndrome [15], Luscan-Lumish syndrome [16,17] and Rabin-Pappas syndrome [16]. These findings suggest that SUPT6H may also contribute to the pathogenesis of NDDs through its role in chromatin regulation. However, *SUPT6H* variants associated with NDDs and animal models for *Supt6* (mouse ortholog) have not been reported.

The Human Gene Mutation Database (HGMD) is a comprehensive repository of genetic variants implicated in human disorders. It encompasses both mutations with confirmed roles in disease causation and sporadic variants identified in human developmental disorders [18]. Upon reviewing HGMD for *SUPT6H* variants, we identified 18 sporadic single-nucleotide variants (SNVs) in human developmental disorders, comprising one nonsense [19] and 17 missense variants [12,19–25]. These findings suggest that *SUPT6H* is an NDD-associated gene, although it has not yet been formally classified as such. We utilized protein modeling to evaluate the impact of the missense variants and assessed their potential to disrupt protein folding, alter surface charge, and modify critical binding interfaces. In addition, we investigated behavioral phenotypes and neuronal loss in a mouse model of *Supt6* deletion, providing insight into its role in NDDs.

## 2. Methods

### 2.1. Mice

All procedures involving animal crossings, biochemical, stereotaxic survival surgery and behavioral experiments were approved by Rutgers University Institutional Animal Care and Use Committee and were in accordance with the National Institutes of Health guidelines. Floxed *Supt6* mice were generated in Taconic-Artemis (Germany) and maintained at Rutgers University. PV-Cre (stock 008069) line [26] was obtained from The Jackson Laboratory (Bar Harbor, ME, USA). We produced the progeny of the *Supt6* cKO^PV^ line (PV-Cre; floxed *Supt6*) by natural breeding or *in vitro* fertilization (IVF) and embryo transfer techniques (Genome Editing Shared Resource, Rutgers University) to provide animals with the same date of birth for the behavioral tests. All mice are of C57BL/6 background. We used both male and female mice in all experiments, except for depression-like or psychiatric disorder–related behavioral assays involving wild-type (WT) and heterozygous cKO^PV^ mice, for which only males were included. Mice were housed 2–5 per cage with a 12 h light/12 h dark cycle and *ad libitum* access to food and water under controlled humidity, temperature (21 ± 2 °C), and air exchange. Mice were assigned to experimental groups based on their genotype. The selection of animal samples from different experimental groups for biochemical analyses was performed randomly and blindly.

### 2.2. Behavioral tests

Mice were tested during the light phase of a 12:12 h light/dark cycle. Mouse behavior was recorded and blindly scored using Ethovision (Noldus).

The open field test (OFT) was performed to measure locomotor activity of the animals. Each animal was placed in the center of a square arena (45 cm × 45 cm) and allowed to freely explore the environment for 10 min. The total distance, the time spent in the center zone, and in the periphery were measured using the Ethovision XT software (VA, USA).

The tail suspension test (TST) was used to measure depressive-like behavior as described [27,28]. Mice were suspended on the edge of a table (elevated ~60 cm above the ground) using tape that was attached 2 cm from the tip of their tails. Prior to suspending the mouse, a small plastic cylinder was placed over the mouse tail to prevent tail climbing. The test was recorded for 6 min and the duration of immobility was analyzed. The animals were considered immobile when they hung down passively and remained completely motionless.

The forced swim test (FST) was used to assess despair-like behavior as described [28,29]. Each mouse was individually placed for 6 min in a cylindrical container filled with water to a depth of 15 cm, with a temperature of 25 ± 1 °C. The cylinder had a diameter of 20 cm and a height of 30 cm. The water was changed between each mouse tested. After the test, the mice were taken out, dried, and returned to their respective home cages. The entire test was video-recorded, and the immobility behavior was subsequently analyzed during the final 4 min of the session. A mouse was considered immobile when it exhibited no movements, or only those minimal movements necessary to keep its head above the water.

The novelty-suppressed feeding test (NSFT) was used to assess anxiety-like behaviors as described [30,31]. The behavioral testing involved placing an animal in a plastic box (50x50x20 cm), the floor of which was covered with a 2 cm layer of wooden bedding. Before the test, the animals were deprived of food for 24 h in their home cages. At the time of the test, two food pellets were placed on a round piece of filter paper with a diameter of 12 cm, which was positioned in the center of the box. The test commenced as soon as the animal was placed in a corner of the box. The time taken for the animal to approach the food pellet and begin feeding (latency to feed) was then recorded, with a maximum recording time of 5 min.

The sucrose preference test (SPT) was used to measure the relative preference of sucrose over water to assess hedonic behavior in mice as described [28,32]. During the 2-day habituation period, mice were given a choice of two water bottles. The following day, bottles were replaced with new bottles containing either water or 1% sucrose solution. The consumption of water and sucrose solution was measured every 24 h for 2 days, and data were averaged. The location of bottles was switched each day. The sucrose preference was represented as percent preference for sucrose: [sucrose consumed/(water consumed + sucrose consumed)] × 100.

The nest-building test (NBT) was used to measure an indicator of health and welfare in experimental mice as described [33,34]. Briefly, mice were single housed, and a cotton nestlet (50 mm × 50 mm square pressed cotton) was provided. Nest condition was scored at 6 h and 24 h after nestlet placement. A score of 1 was assigned for a nestlet un-touched, 2 was for a nest partially torn up, and 3 was for a nestlet that had been almost entirely shredded, although there was no clear nest area. Only when the nestlet was entirely shredded and a nest area established, a score of either 4 (nest was flat) or 5 (perfect crater with walls higher than the body height of the mouse) was given.

Sucrose splash test (SST) was performed to measure self-care behavior as described [35]. The mice were placed individually in a clear observation cage without food. Subsequently, the dorsal fur of each mouse was sprayed with a 10% sucrose solution. The presence of this sucrose solution dirties the fur, which typically stimulates grooming behavior in mice. The latency to the first grooming and the duration of the grooming were recorded during a 5-min session. An increase in latency to start grooming and a decrease in the duration of this self-grooming behavior are interpreted as depression-like behavior.

### 2.3. Immunohistochemistry (IHC)

After the behavior tests, the mice were anesthetized with CO2 and intracardially perfused with phosphate-buffered saline (PBS), followed by 4% paraformaldehyde in PBS. The brains were removed from the skulls and post-fixed overnight at 4 °C. Then, the brains were incubated with a solution of 30% of sucrose diluted in PBS. Brain sections of 20 μm were cut using a cryostat (Leica, CM3050S), and the slices were stored at −80 °C until the day of immunohistochemistry analysis.

All staining between groups was done with the same solution mix of blocking buffer and antibodies. Slide-mounted sections were washed in PBS and subsequently incubated in blocking buffer (0.5% Triton X-100, 5% normal goat serum, in PBS) for ~2 h at room temperature. Sections were then incubated overnight at 4 °C in the primary antibodies diluted in blocking buffer. The following primary antibodies were used to detect PV, Supt6 or GFP. Mouse anti-PV (1:100, Swant, PV235), rabbit anti-SUPT6H (1:200, Bethyl, A300-801A) and rabbit anti-GFP (1:1000, Abcam, ab290). DAPI counterstaining was used to visualize the nuclei. After incubation, sections were washed three times in PBS and incubated with Alexa-fluor-conjugated secondary antibodies: goat anti-mouse 488 (Invitrogen, 1:1000), and goat anti-rabbit 570 (Invitrogen, 1:1000). After secondary incubation, sections were washed in PBS three times and mounted on glass slides with Vectashield Hardset with Dapi (Vector Labs) for microscopy. Confocal images were obtained on a Zeiss LSM 800 confocal imaging system (Carl Zeiss Microscopy). Gain, exposure time, and all other related settings were constant throughout each experiment. All image groups were processed in parallel using Fiji.

### 2.4. RNA fluorescent in situ hybridization (RNAscope)

Mice were deeply anesthetized using CO2 and transcardially perfused with PBS, followed by 4% paraformaldehyde (PFA) in PBS. Brains were post-fixed in 4% PFA overnight at 4 °C, and then cryoprotected using 30% sucrose in PBS for at least 24 h, followed by freezing and embedding in Tissue Tek OCT medium (Sakura Finetek USA Inc., CA, United States). A cryostat was used to collect 15-μm-thick coronal sections, mounted on slides, and stored at −80 °C. Sections were fixed in 4% PFA for 15 min, dehydrated in serial ethanol concentrations (50, 70, and 100%), and processed with the RNAscope Multiplex Fluorescent Assay v2 (323100, RNAscope, Advanced Cell Diagnostics, Inc., CA, United States). Sections were hybridized with a mixture of selective probes for parvalbumin (Mm-Pvalb, 421931) and supt6 (Mm-Supt6-O1-C3, 1166741). Sections were then counterstained with DAPI and coverslipped. Confocal images were obtained on a Zeiss LSM 800 confocal imaging system (Carl Zeiss Microscopy). Gain, exposure time, and all other related settings were constant for each quantified image. To generate a projection image for each PV cell, each set of stack projections was z-stacked with maximum intensity using Fiji. To minimize the Supt6 puncta from other cell types, regions-of-interest (ROIs) were made around only dense PV mRNA puncta to capture the PV-expressing cytoplasmic area of PV neurons in the hippocampus. These ROIs were then used for puncta count analysis using Fiji.

### 2.5. Stereotaxic surgery

All stereotaxic surgeries were performed under aseptic conditions using an anesthetic as described previously [36]. Mice were anesthetized with 2% isoflurane and maintained at 1–1.5% isoflurane during the surgery. AAV5-hSyn-GFP and AAV5-hSyn-Cre-GFP were obtained from UNC Vector Core. Viruses (300 nL/side) were injected bilaterally into the hippocampus (AP- 2 ML + 1.5 DV- 1.75 mm relative to bregma) with a 2 mL Hamilton Neuros Syringe at a speed of 0.1 μL/min. The needle was left for an additional 10 min and then slowly withdrawn. Mice were monitored for 48 h to ensure full recovery from the surgery. Experiments commenced 3 weeks after stereotaxic surgery to allow optimal expression of AAVs.

### 2.6. Timm staining

Mossy fiber sprouting was measured by Timm staining using the FD Rapid TimmStain Kit (FD Neurotechnologies, Inc.). WT and *Supt6* cKO^PV^ mice were deeply anesthetized with CO2 and then perfused intracardially with the perfusate provided in the kit (freshly made by mixing equal volumes of perfusates A and B) for 7 min and immediately followed by 10% buffered neutral formalin. Brains were removed from the skull and postfixed in the same fixative for 24 h at 4 °C. Subsequently, brains were transferred in 0.1 M PBS containing 30% sucrose and stored at 4 °C for 48 h before freezing. Sections (40 μm thickness) were cut on a cryostat and mounted on Superfrost Plus slides. After air-drying, sections were stored at −20 °C before processing. Slides containing 3 sections/animal were then processed with the staining steps as instructed in the user manual for the FD Rapid TimmStain Kit (PK 701, Version 2012-01). Finally, slides were coverslipped with Permount mounting medium and stored in the fridge until imaging with a Leica fluorescent microscope using only the light mode. The relative optical density (ROD) was calculated with Fiji, as described previously [37].

## 3. Results

### 3.1. Identification of coding variants in SUPT6H in developmental disorders

**Fig. 1.**
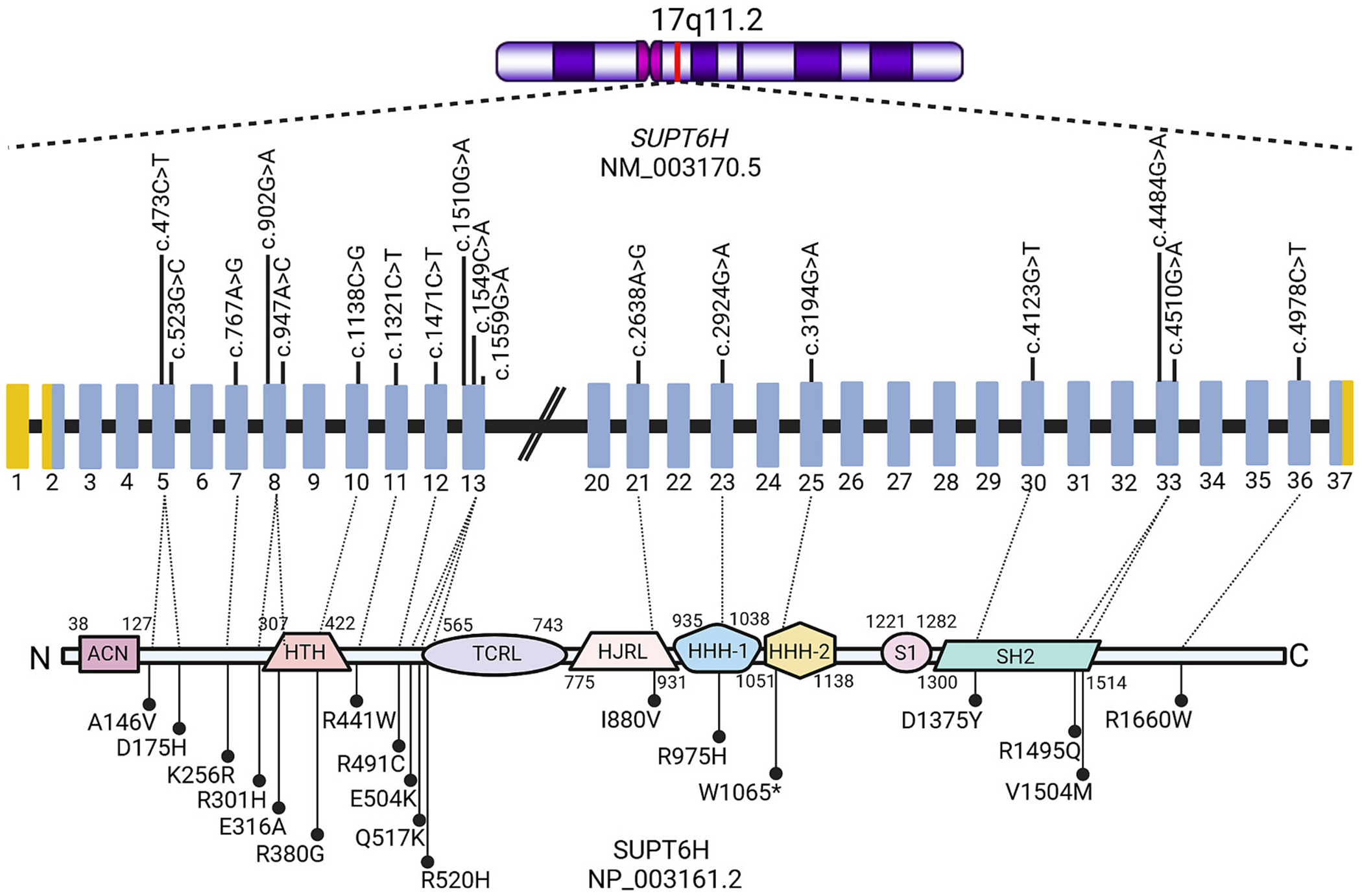
Localization of sporadic variants in *SUPT6H* at 17q11.2. The upper panel shows the reported 18 intragenic variants within exons of *SUPT6H* transcript variant 2 (NM_003170.5) Exons are depicted as blue boxes, connected by a thick horizontal black line representing introns, while the yellow boxes indicate the 5′- and 3′- UTRs. The lower panel illustrates the domain structure of the SUPT6H protein isoform NP_003161.2 (aa 1-1726), with dotted black lines linking the reported mutations to their corresponding amino acids. Functional domains are represented by boxed and circular shapes as defined by InterPro annotations (https://www.ebi.ac.uk/interpro/): ACN: acidic N-Terminal (aa 38-127), HTH: helix-turn-helix DNA-binding domain (aa 307-422), TCRL: Tex central region-like domain (aa 565-743), HJRL: Holliday-junction resolvase-like domain (aa 775-931), HHH-1: helix-hairpin-helix like domain 1 (aa 935-1038), HHH-2: helix-hairpin-helix like domain 2 (aa 1051-1138), S1: S1 RNA binding domain (aa 1227-1282), SH2: Src Homology 2 domain (aa 1300-1514).

We identified 18 sporadic SNVs, comprising one nonsense [19] and 17 missense [12,19–25] variants, from multiple large-scale sequencing cohorts of individuals with human developmental disorders (Fig. 1 and Table 1). We analyzed the effects of the various SNVs on the structure at an atomic level. Molecular modeling of these 17 variants was conducted using the crystal structure of the SH2 domain of SUPT6H comprising residues 1338-1519 (PDB code 6GME) [6], the cryo-electron microscopy (cryo-EM) structure of the human RNA polymerase II–transcription-coupled DNA repair complex containing the SPT6 (residues 284–1287) fragment as a component (PDB code 7OOP) [38], and the three-dimensional structural model of the full-length form obtained from the Alphafold protein structure database (https://alphafold.ebi.ac.uk; Q7KZ85).

**Fig. 2.**
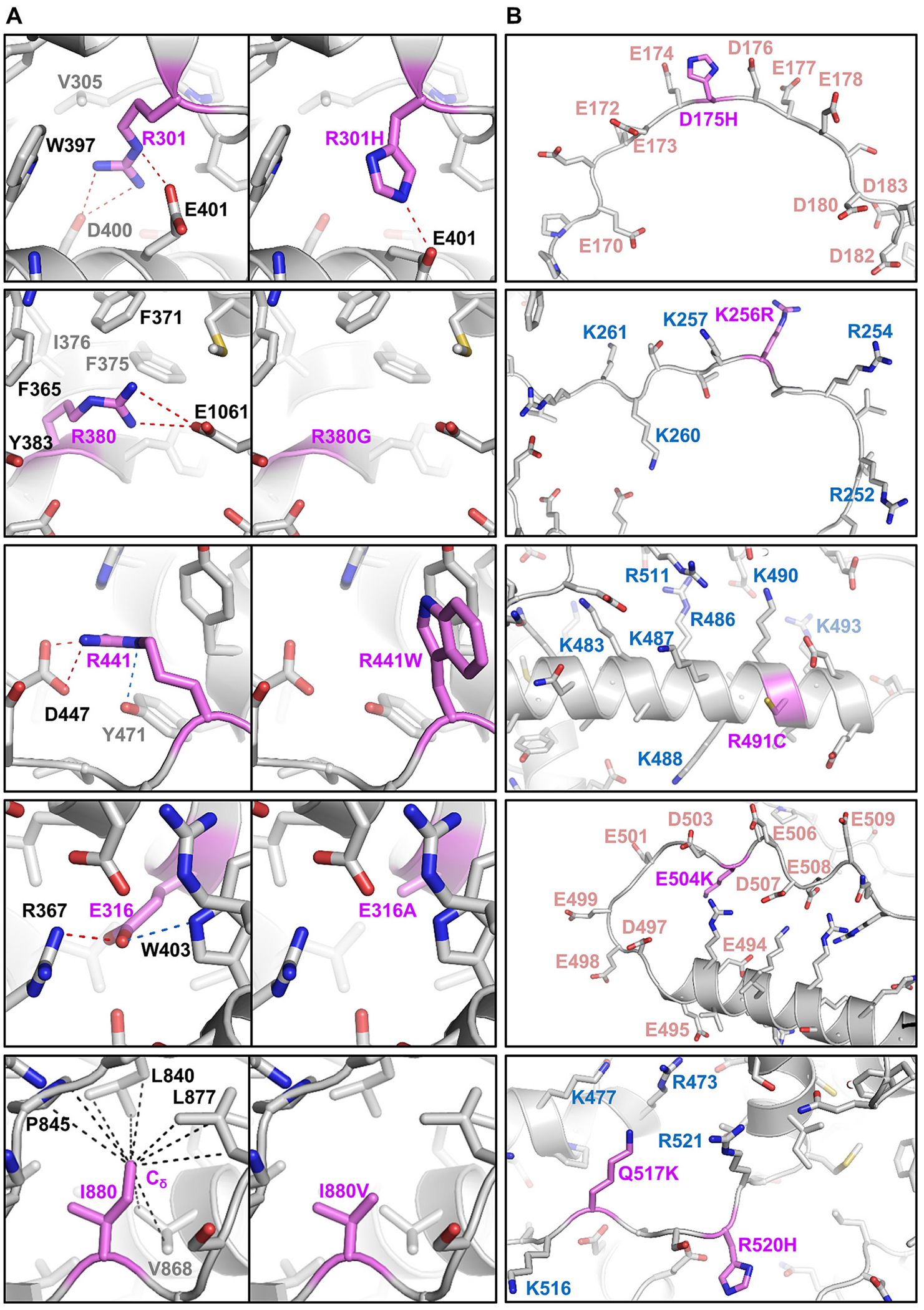
Molecular modeling of ten variants of *SUPT6H*. (A) Protein misfolding-inducing mutations. Left, wild-type; right, the indicated mutant form. The residues targeted to be substituted are represented in violet and labeled. Residues associated with intermolecular interaction with the substitution-targeted residues are also labeled. Dashed lines indicate carbon–carbon contacts (black), ionic interactions (red), and hydrogen bonds (blue). (B) Mutations found in the charged clusters. The substituted residues are represented in violet and labeled. Residues consisting of the charged clusters are labeled in blue (arginine and lysine, positively charged) or in pale pink (aspartate and glutamate, negatively charged).

We first found that seven substitutions are expected to cause protein misfolding: Arg301His, Glu316Ala, Arg380Gly, Arg441Trp, and Ile880Val severely, while Ala146Val and Val1504Met slightly. Arg301, Arg380, and Arg441 play a key role in sustaining the protein folding *via* a combination of intramolecular electrostatic interactions (Arg301 with Asp400 and Glu401; Arg380 with Glu1061; Arg441 with Asp447), hydrogen bonds (Arg441 with Tyr471), and hydrophobic interactions (Arg301 with Val305 and Trp397; Arg380 with Phe365, Phe371, Phe375, Ile376, and Tyr383; Arg441 with Tyr471) (Fig. 2A, row 1–3). Likewise, the side chain carboxyl group of Glu316 is shown to attract the guanidinium group of Arg367 by ionic interaction and the indole group of Trp403 by hydrogen bonding (Fig. 2A, row 4), and the C_δ_ atom of Ile880 mediates intramolecular hydrophobic interaction *via* 10 intramolecular carbon–carbon contacts (<5.0 Å) with the side chain hydrocarbon atoms of Leu840, Pro845, Val868, and Leu877 (Fig. 2A, row 5). All these contacts are absent in the Arg301His, Glu316Ala, Arg380Gly, Arg441Trp, or Ile880Val-substituted models (Fig. 2A, second column), likely leading to a severe impairment of the interior protein folding of SUPT6H and its functionality. In contrast, A146V and V1504M are predicted to only slightly affect protein folding, as the substituted residues (valine/methionine) are bulkier than the original amino acids (alanine/valine) and therefore able to push adjacent residues to avoid steric hindrance (Supplementary Fig. 1A).

Next, structural analysis showed that the rest of the variants occur at surface-exposed residues and thus they might alter surface charge and shape of SUPT6H. Especially, SUPT6H contains several “charged” clusters, in which negatively (aspartate and glutamate) or positively (lysine and arginine) charged residues are intensively concentrated. Based on the previous structural investigations [39–41], we suppose that such charged regions play a substantial role in association with other proteins or nucleic acids. We found six variants that occur at such regions, including Asp175His, Lys256Arg, Arg491Cys, Glu504Lys, Gln517Lys, and Arg520His (Fig. 2B). These substitutions are expected to alter the surface charge and shape of the clusters, leading to interference with proper contacts of SUPT6H with its binding partners. The remaining four variants, Arg975His, Asp1375Tyr, Arg1495Gln, and Arg1660Trp, are also expected to affect surface charge and shape of SUPT6H (Supplementary Fig. 1B). While these variants may affect binding interactions mediated by SUPT6H, their precise structural and functional consequences remain to be elucidated. Collectively, our structural analysis suggests that these variants affect either SUPT6H protein folding (Ala146Val, Arg301His, Glu316Ala, Arg380Gly, Arg441Trp, Ile880Val, and Val1504Met) or surface charge or surface features putatively required for binding interactions (Asp175His, Lys256Arg, Arg491Cys, Glu504Lys, Gln517Lys, Arg520His, Arg975His, Asp1375Tyr, Arg1495Gln, and Arg1660Trp).

### 3.2. Generation of a knockout (KO) mouse model for Supt6

**Fig. 3.**
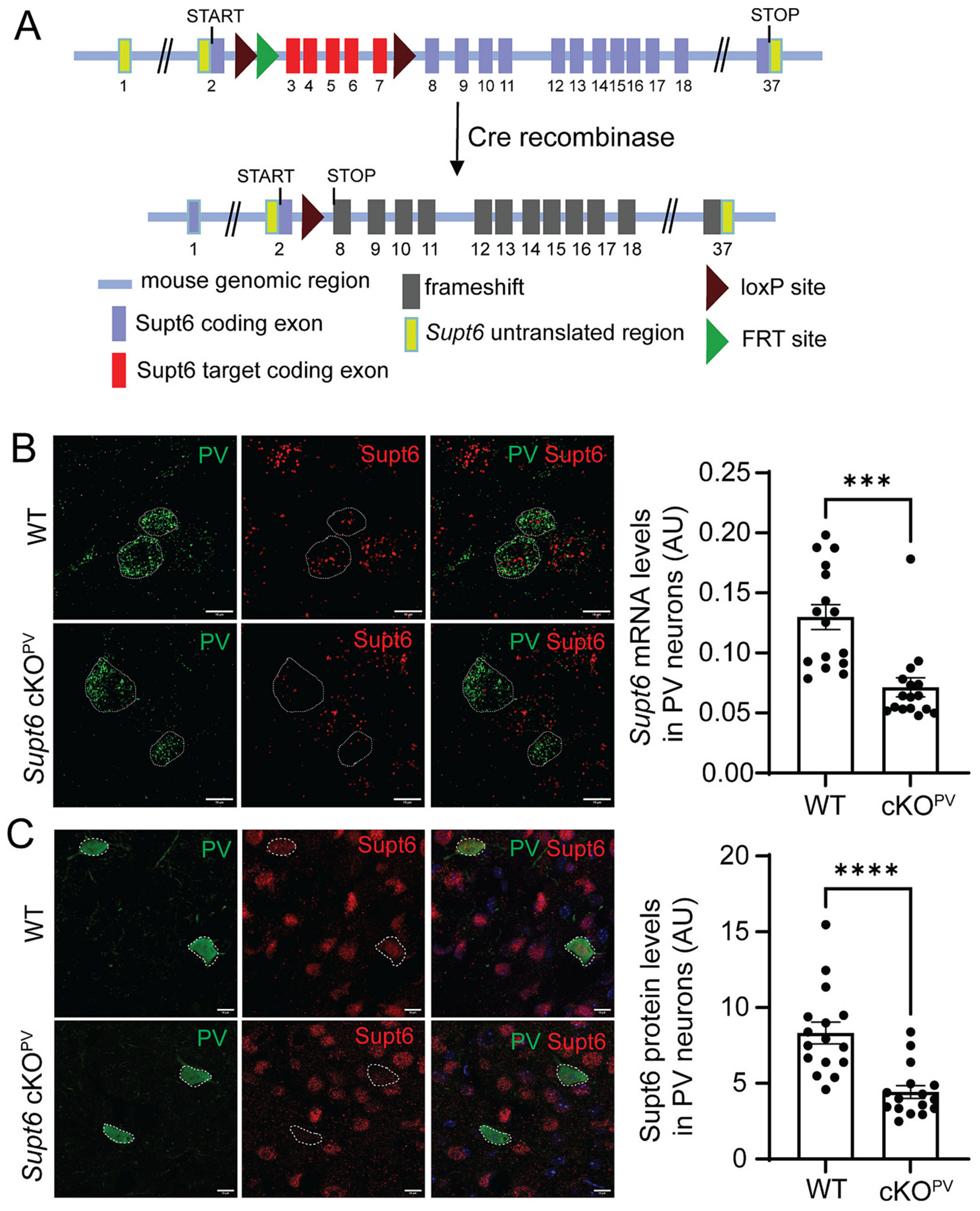
Generation of PV neuron-specific *Supt6* KO mice. (A) Targeting strategy. Exons 3–7 of the *Supt6* gene are flanked by loxP sites, and the deletion of exons by a Cre-recombinase creates a frameshift in downstream exons, generating an early STOP codon. (B) Expression of Supt6 in PV interneurons. Representative images of PV (*Pvalb***)** and *Supt6* mRNA expression visualized by RNAscope in the layer 5 of the cortex (left). *Supt6* mRNA puncta per PV cells were measured and normalized to PV neuron area from wild-type and *Supt6* cKO^PV^ mice. (**C**) Representative images of immunohistochemistry of Supt6 in PV neurons in the layer 5 of the cortex. The integrated optical density of Supt6 immunostaining in PV cells was measured. WT (Supt6^fl/fl^; PV-Cre^+/+^, *n* = 4 animals, 3–4 sections per animal) *versus* Supt6 cKO (Supt6^fl/fl^; PV-Cre^Cre/+^, n = 4 mice, 4–5 sections per animal). Scale bar, 10 μm. Data are presented as mean ± SEM. Individual data points are brain sections. ***p < 0.001, ****p < 0.0001, two-tailed unpaired Student’s *t*-test.

In order to establish the physiological relevance of the loss-of-function of *SUPT6H*, we generated a floxed *Supt6* line by inserting loxP sites to flank exons 3–7 (Fig. 3A). First, we crossed floxed *Supt6* mice with Cre deleter mice, which express Cre recombinase in the whole body, including germ cells. However, we were unable to obtain any viable heterozygous KO mice. Specifically, when we crossed Cre deleter^Cre/+^ mice with floxed Supt6^*fl/*+^ mice, among over 40 pups, ~25% of them were [Cre deleter^*Cre/*+^; floxed Supt6^+/+^], ~42% were [Cre deleter^+/+^; floxed Supt6^*fl/*+^], and ~33% were [Cre deleter^+*/*+^; floxed Supt6^+*/*+^]. However, none of the pups was [Cre deleter^*Cre/*+^; floxed Supt6^*fl/*+^], implicating embryonic lethality of heterozygous KO mice.

To investigate the effect of the loss-of-function of Supt6 on neuronal development, we decided to generate parvalbumin (PV)-expressing GABAergic interneuron-specific conditional KO (cKO^PV^). In rodents, PV expression in these neurons starts at the second postnatal week [42,43]. PV-expressing interneurons are the largest class, constituting roughly 40% of all neocortical GABAergic neurons [44,45] and about 25% of all hippocampal GABAergic neurons [46,47]. Since GABAergic interneurons constitute ~10–20% of all neuronal cell populations [47], we anticipated that *Supt6* cKO^PV^ would survive and provide a model to investigate the effects of the *Supt6* deletion on neurons.

Pups homozygous for the floxed *Supt6* allele and heterozygous for the PV-Cre allele (PV-Cre^*Cre/*+^; floxed Supt6*^fl/fl^*) were born as expected. However, we found only a ~50% reduction of *Supt6* mRNA by RNA *in situ* hybridization (RNAscope) (Fig. 3B) and a ~50% reduction in Supt6 protein levels measured by immunohistochemistry (Fig. 3C). AAV-mediated expression of Cre recombinases in the hippocampus of homozygous floxed *Supt6* (Supt6*^fl/fl^*) mice also significantly but not completely reduced the levels of Supt6 (Supplementary Fig. 2). Inefficient recombination of some flanked loxP sites by Cre recombinases is known [48,49], and this may explain the partial reduction of Supt6 in PV neurons in the homozygous *Supt6* cKO^PV^ mice.

### 3.3. Homozygous Supt6 cKO^PV^ pups display severe motor defects

**Fig. 4.**
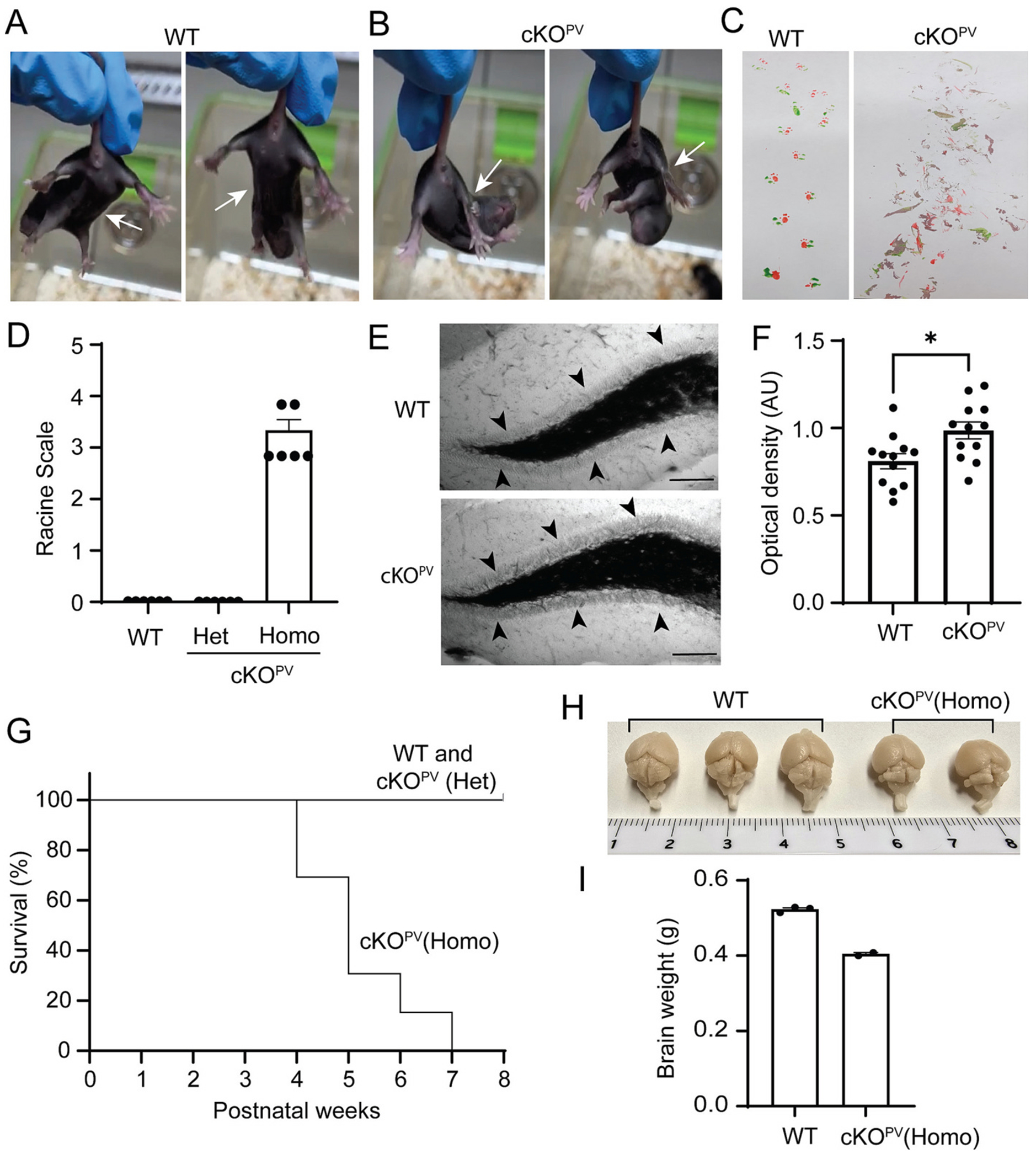
Motor defects and behavioral seizures in *Supt6* cKO^PV^. (A, B) WT mice display normal posture (A), but homozygous *Supt6* cKO^PV^ mice (B) display a phenotype of limb-clasping. (C) Gait analysis shows the walking pattern in WT (left) and homozygous *Supt6* cKO^PV^ mice (right). Red paint, forelimb; green paint, hindlimb. (D) Behavioral seizure scores in WT control and heterozygous and homozygous *Supt6* cKO^PV^ mice. *n* = 6 mice per group. (E) Representative Timm Staining in WT and homozygous *Supt6* cKO^PV^ mice. Scale bar, 50 μm. (F) Quantification of the optical density of the inner molecular layer of the dentate gyrus. *n* = 3 mice per group, and 4 sections per mouse. Data are presented as mean ± SEM, and individual data points are depicted. *p < 0.05, Two-tailed unpaired Student’s *t*-test. (G) Survival curve for homozygous *Supt6* cKO^PV^ (*n* = 13), heterozygous cKO^PV^ (*n* = 46) and WT mice (*n* = 47). (H, I) Brain images of WT (n = 3) and homozygous *Supt6* cKO^PV^ (*n* = 2) littermates at postnatal day 42 (H). Quantification of brain weight (I).

Our first discovery was that homozygous, but not heterozygous, *Supt6* cKO^PV^ mice displayed severe motor defects (Supplementary Video 1). In a limb-clasping test [50], when mice are gently lifted by their tails, WT mice tend to extend their hindlimbs away from their abdomen when suspended (Fig. 4A). However, *Supt6* cKO^PV^ mice show an abnormal response by retracting or “clasping” their hindlimbs towards their body (Fig. 4B). This abnormal degree of limb retraction indicates motor impairment or neurological dysfunction. When we analyzed walking pattern (gait analysis), WT mice displayed normal walking with even participation of the forelimb (red paint in Fig. 4C) and hindlimb (green paint). However, *Supt6* cKO^PV^ mice displayed a severe limp and difficulty moving forward (Supplementary Video 1 and Fig. 4C). In addition, young *Supt6* cKO^PV^ pups often display tremor (Supplementary Video 2). Homozygous *Supt6* cKO^PV^ mice, but not heterozygous cKO^PV^ mice, also exhibit a behavioral seizure phenotype, such as forelimb clonus with lordotic posture or rearing and falling [51] (Fig. 4D). We also found that Timm staining shows a modest but significant increase of mossy fiber sprouting in the dentate gyrus in *Supt6* cKO^PV^ compared to WT control brains, supporting the seizure activity in *Supt6* cKO^PV^ mice (Fig. 4E and F). The enhanced mossy fiber sprouting represents the growth of granule cell axons into their own dendritic field in the inner molecular layer, indicating the epileptic brain’s increased excitability in *Supt6* cKO^PV^ mice.

Due to severe neurological defects, mortality in homozygous *Supt6* cKO^PV^ mice was observed between 4 and 7 postnatal weeks (Fig. 4G). The brain size of homozygous *Supt6* cKO^PV^ mice was also smaller than that of their wild-type (WT) littermates (Fig. 4H, I). The body size of homozygous *Supt6* cKO^PV^ mice was smaller compared to WT or heterozygous *Supt6* cKO^PV^ mice (Fig. 4A, B, and Supplementary Videos 1 and 2).

### 3.4. Heterozygous Supt6 cKO^PV^ mice display psychiatric disorder-related behavioral phenotypes

**Fig. 5.**
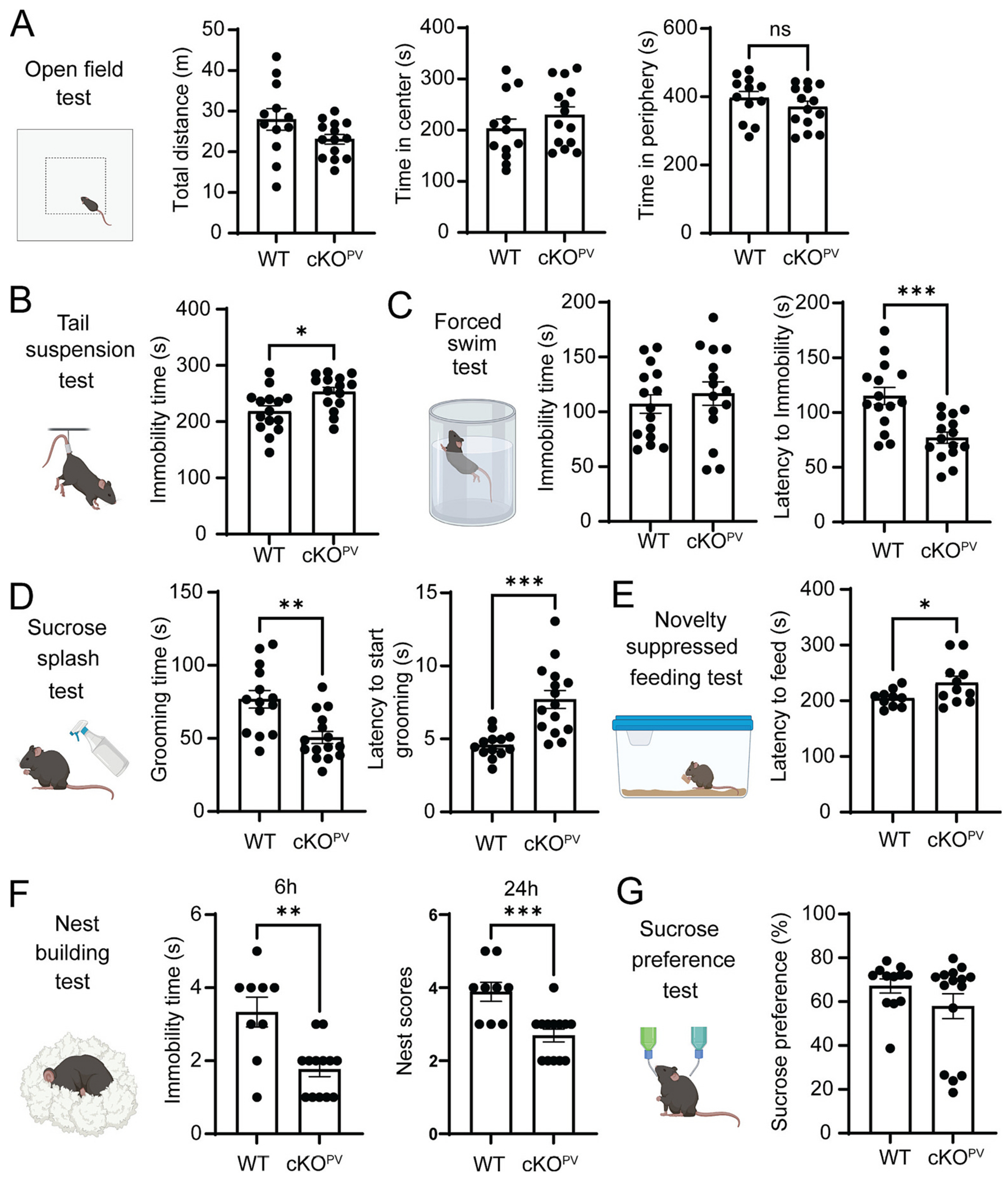
Heterozygous *Supt6* cKO^PV^ mice display behavioral abnormalities related to psychiatric disorders. (A) Locomotor activity in the OFT. Total distance (left), time in the center (middle), and time in the periphery (right). (B) Total immobility time in the TST. (C) Total immobility time (left) and the latency to immobility (right) in the FST. (D) Total grooming time (left) and the latency to start grooming in the SST. (E) Latency to start feeding in the NSF. (F) Nesting scores 6 h (left) and 24 h (right) after single housing. (G) Sucrose preference in the SPT. WT mice (*n* = 12–15 males) and heterozygous *Supt6* cKO^PV^ mice (n = 13–15 males). *p < 0.05, **p < 0.01, ***p < 0.001. Two-tailed unpaired Student’s *t*-test.

Previously, Supt6 was identified as a binding partner of the multi-functional protein S100a10 (also called p11) [31], alterations of which have been implicated in the etiology of major depressive disorder and the actions of antidepressants [52–54]. For example, alterations of PV neuronal firing in the dentate gyrus underlie chronic social defeat-induced social avoidance [55]. p11 is expressed in PV neurons [31] and a genetic KO of p11 gene (*S100a10*) or its binding partners in PV neurons alters depression-like behavior [55,56]. Because homozygous *Supt6* cKO^PV^ mice displayed severe motor defects and behavioral seizures, we were not able to analyze depression-like behavior of them. However, heterozygous *Supt6* cKO^PV^ mice displayed normal locomotor activity comparable to WT control group in the open field test (OFT) (Fig. 5A). Thus, we examined several depression-like or neuropsychiatric disorder-related behaviors in these mice.

Heterozygous *Supt6* cKO^PV^ mice display depression-like behavior by showing increased immobility in the tail suspension test (TST) (Fig. 5B). In the forced swim test (FST), *Supt6* cKO^PV^ mice display a shorter latency for immobility or faster despair, although total immobility time did not differ significantly from that of WT controls (Fig. 5C). *Supt6* cKO^PV^ mice also showed reduced total grooming time and increased latency to start grooming in the sucrose splash test (SST), supporting depression-like behavior (Fig. 5D). Furthermore, *Supt6* cKO^PV^ mice showed increased latency in the novelty suppressed feeding test (NSFT) (Fig. 5E). The increased latency to feed in the NSFT is due to depression-like behavior or increased anxiety. Because *Supt6* cKO^PV^ mice do not show thigmotaxis (the tendency to remain close to the walls of an arena) in the OFT (Fig. 5A), the result in the NSFT may reflect the depression-like phenotype in *Supt6* cKO^PV^ mice. Nest building is an innate behavior in rodents, and it is impaired in several neurological disorder- and psychiatric disorder-related rodent models [34,57,58]. We also observed poor nesting scores in the *Supt6* cKO^PV^ group compared to the control group at 6 h and 24 h after single housing (Fig. 5F). These results together indicate a cluster of depression-like or psychiatric disorder-related behavioral phenotypes. However, *Supt6* cKO^PV^ mice did not display an anhedonia-like phenotype in the sucrose preference test (SPT) (Fig. 5G).

### 3.5. PV neuronal loss in Supt6 cKO^PV^ mice

**Fig. 6.**
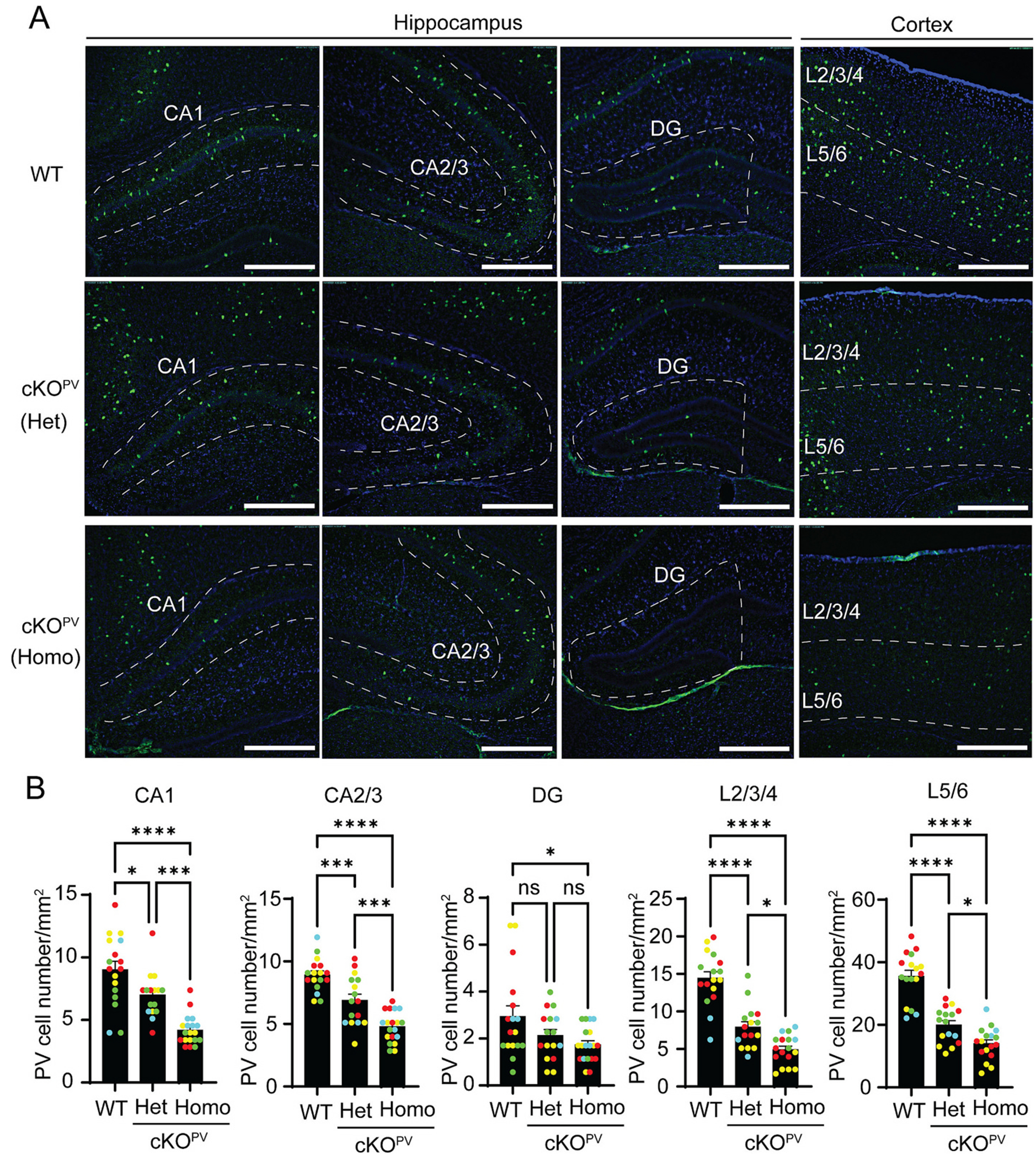
A decrease in the number of PV neurons in *Supt6* cKO^PV^ mice. (A) Representative images of hippocampal areas and primary/secondary motor cortical areas from WT, heterozygous (Het) or homozygous (Homo) *Supt6* cKO^PV^ mice. The regions for PV cell counting are outlined with dotted lines. (B) Significant reduction of PV-expressing neurons in the CA1, CA2/3, dentate gyrus (DG), cortical layer 2/3/4 and cortical layer 5/6 in heterozygous and homozygous cKO^PV^ mice compared to WT control mice. Scale bar, 400 μm. Data are mean ± SEM *p < 0.05, ***p < 0.001, ****p < 0.0001, n = 4 mice. Individual data points are brain sections. One-way ANOVA, *Post-hoc* Bonferroni’s multiple comparisons.

Clinically, a loss of PV interneurons is common in the brains of individuals affected by NDDs, including ASD [59], schizophrenia [60] and epilepsy [61,62]. We therefore analyzed the number of PV-expressing neurons. We found that the number of PV interneurons is significantly decreased in both heterozygous and homozygous *Supt6* cKO^PV^ mice across hippocampal and cortical brain regions (Fig. 6A and B). The decrease in the number of PV neurons in heterozygous *Supt6* cKO^PV^ mice is modest but significant. A bigger reduction was observed in homozygous *Supt6* cKO^PV^ mice. These results suggest that Supt6 is required for PV neuronal survival, and Supt6 reduction in PV neurons results in PV neuronal loss and behavioral defects.

## 4. Discussion

Next-generation sequencing has produced vast genomic datasets from large cohorts of individuals with NDDs, revealing many candidate genes associated with NDDs. However, the available datasets have not been fully explored, and strategies to effectively utilize these genetic variants from such large datasets are limited. This highlights the need for systematic data mining and targeted functional studies to validate the roles of novel candidate genes in NDD pathogenesis.

SUPT6H is a key transcriptional regulator with dual functions as an elongation factor [5–7] and histone chaperone [7,8]. It stabilizes RNA polymerase II during transcription elongation and preserves chromatin integrity to ensure proper gene expression. These functions are essential for neuronal development and function. Notably, disruptions in genes involved in transcriptional regulation and chromatin remodeling have been linked to various NDDs, such as ASD, intellectual disability, and epilepsy [1].

To date, no studies have reported coding variants in *SUPT6H* associated with NDDs. In this study, we analyzed 18 sporadic *SUPT6H* SNVs, including one nonsense [19] and 17 missense variants [12,19–25], identified from large-scale next-generation sequencing studies of individuals with developmental disorders (Fig. 1 and Table 1). From the large cohort studies, the detailed clinical phenotypes for individual patients harboring SUPT6H variants are not available. Consequently, the only recurrent features documented are developmental delay, NDD and/or ASD, which are consistent across most cases and align with typical neurodevelopmental presentations. Among the 18 identified *SUPT6H* variants, three (cases #2, #5, and #8 in Table 1) were found in individuals with congenital heart disease (CHD). NDD represents the most common comorbidity in individuals with CHD, and many affected patients exhibit developmental delay or cognitive impairments [63–66]. In addition, one case (case #11 in Table 1) is orofacial clefting. Notably, individuals with orofacial clefts, including cleft lip with or without palate and cleft palate only, have a significantly increased risk of neurodevelopmental disorders, including a higher prevalence of ASD, attention-deficit/hyperactivity disorder (ADHD), intellectual disabilities, and language delays compared to the general population [67–69]. Variant interpretation was performed according to The American College of Medical Genetics and Genomics (ACMG) guidelines [70], with population data used to assess the prevalence of these variants in the general population [71]. This approach enabled the identification of rare or novel variants (Table 1). To further support the pathogenicity assessment, we used the Combined Annotation Dependent Depletion (CADD) score, a comprehensive tool that integrates multiple functional annotations [72], classifying variants with a CADD score >20 as pathogenic or likely pathogenic (Table 1). Among the 18 heterozygous variants we analyzed, 17 had a CADD score greater than 20, with 16 classified as likely pathogenic and one nonsense variant [19] classified as pathogenic based on ACMG interpretations. The missense variant p. Ile880Val [12,19,25], which had a CADD score of 18.65, was still interpreted as likely pathogenic according to ACMG guidelines (Table 1). Interestingly, eight missense variants are in functional domains, likely disrupting the functional activity of SUPT6H or its ability to interact with other proteins involved in chromatin remodeling (Fig. 1).

Our *in silico* predictions suggest that these variants may significantly alter the function of SUPT6H. Among them, thirteen SUPT6H variants were present in the Genome Aggregation Database (gnomAD, a dataset from healthy subjects) at low frequencies in the heterozygous state [73] (Table 1). In fact, seven truncating alleles in *SUPT6H* (p.Tyr229Ter, p.Glu382Ter, p.Tyr425Ter, p.Lys738Ter, p.Trp752Ter, p.Tyr1141Ter, and p.Gln1637Ter) were listed in the gnomAD [73]. Each of these alleles is extremely rare, observed only once among more than 1.6 million alleles (allele frequency is ~6 × 10^−7^). Therefore, the existence of ultra-rare truncating alleles in population databases does not refute pathogenicity. Mildly affected carriers may be misclassified as healthy controls, and ascertainment biases can under-represent neurobehavioral symptoms. For example, established autosomal dominant, haploinsufficient NDD genes, such as *PHF21A* [74,75] and *NRXN1* [76,77], also harbor rare nonsense variants in gnomAD. Importantly, constraint metrics strongly support a haploinsufficiency mechanism of *SUPT6H*. The pLI score is 1.0 in gnomAD, indicating a profound scarcity of loss-of-function variants in the control population. Particularly, the intolerance to truncating variants suggests that dosage reduction is deleterious. Of note, among the 18 *SUPT6H* variants reported in the literature, Asp175His, Lys256Arg, Arg380Gly, Gln517Lys, and Trp1065Ter were not found in the gnomAD, indicating they may be pathogenic due to their more pronounced effects on SUPT6H function. Establishing a reliable model [78] for the *SUPT6H* variants and characterizing the functional activity of *SUPT6H* variants *in vivo* would be critical.

It was experimentally shown that an altered RNA polymerase II elongation rate can result in embryonic lethality [79]. IWS1 and SUPT6H play crucial roles in embryonic genome activation, lineage specification, and histone modification during early mouse development. Ablation of *Iws1* in mice resulted in early embryonic lethality [80]. Since we could not obtain pups with a heterozygous or homozygous *Supt6* null allele, we used a minor neuronal population-specific deletion approach for *Supt6*. Because the PV gene promoter becomes active from the second postnatal week [42,43] and PV interneurons have been implicated in psychiatric disorders and epilepsy [81], we decided to generate PV neuron-specific *Supt6* KO mice. To the best of our knowledge, this is the first mouse model featuring a *Supt6* KO in a specific neuronal type.

We observed only partial deletion of *Supt6* in the homozygous floxed *Supt6* mice carrying a heterozygous PV-Cre allele. Because the PV-Cre line used in this study is a well-established and popular line [26,28,55,56], the recombination efficiency of only ~50% in floxed mice is unusual (Fig. 3). Similarly, AAV-mediated Cre recombinase expression in the hippocampus resulted in incomplete *Supt6* deletion (Supplementary Fig. 2). Of note, we have previously used this same PV-Cre line in several studies [28,55,56] and consistently observed much higher recombination efficiency. Thus, the partial deletion is likely due to the inefficient recombination at the loxP sites flanking *Supt6* exons 3–7 by the PV promoter-driven Cre recombinases [48,49]. Despite the partial deletion, *Supt6* cKO^PV^ mice displayed a reduced number of PV neurons in the hippocampus and cortex (Fig. 6). A ~50% reduction of Supt6 in PV neurons results in severe motor defects, reduced brain size, and spontaneous seizures, suggesting Supt6 as a pivotal regulator of neuronal development and survival. PV inhibitory neurons are crucial for maintaining the excitatory-inhibitory (E/I) balance, a hallmark feature of NDD pathophysiology [82]. Dysregulation of Supt6-mediated transcriptional regulation in PV neurons may result in alterations in neuronal firing and E/I imbalance within the neural circuits [83,84], and ultimately influence phenotypes in *Supt6* cKO^PV^ mice. In this context, an alternative mechanism underlying the loss of PV neurons in *Supt6* cKO^PV^ might be seizure-related overactivation of excitatory neurons rather than the primary effect of *Supt6* cKO^PV^ on PV neuronal survival.

Heterozygous *Supt6* cKO^PV^ mice also display a modest but significant reduction in the number of PV neurons, accompanied by behavioral phenotypes related to psychiatric disorders compared to WT mice. Previously, we identified SUPT6H as an interactor of p11, a protein whose alterations are highly implicated in the etiology of major depressive disorder and antidepressant responses [31,52–54]. Along with ANXA2, p11 constitutes the SUPT6H protein complex, which is involved in epigenetic regulation and the expression of pluripotency factors in breast cancer stem cells [85]. p11 and ANXA2 bind to the C-terminal region of SUPT6H (amino acids 1650–1726) [54]. The Trp1065Ter variant of *SUPT6H* found in NDD patients may disrupt this interaction with p11 and ANXA2. SUPT6H also directly interacts with IWS1, an RNA polymerase II-associated elongation factor [86,87]. A recent GWAS study implicates *IWS1* as a potential gene associated with suicide attempts and mood disorders [88]. Altered PV neuronal firing in the dentate gyrus underlies avoidance behavior induced by chronic social defeat stress [55]. p11 is expressed in PV neurons [31], and KO of p11 or its binding partners, such as mGluR5, SMARCA3 or Ahnak, in PV neurons modulates depression-like behavior [28,55,56]. Correspondingly, heterozygous *Supt6* cKO^PV^ mice display depression-like behavior and impaired nest-building ability (Fig. 5), implicating that *SUPT6H* variants may contribute to psychiatric symptoms in NDDs. Of note, we analyzed only males in the depression-like or psychiatric disorder-related behavioral tests, although both male and female mice were used in all other experiments, and comparable phenotypes were observed in both sexes. Given known sex differences in PV neuronal function [89,90] and psychiatric disorder-related behavior [91,92], behavioral phenotypes of heterozygous *Supt6* cKO^PV^ females should be evaluated in future studies.

**Fig. 7.**
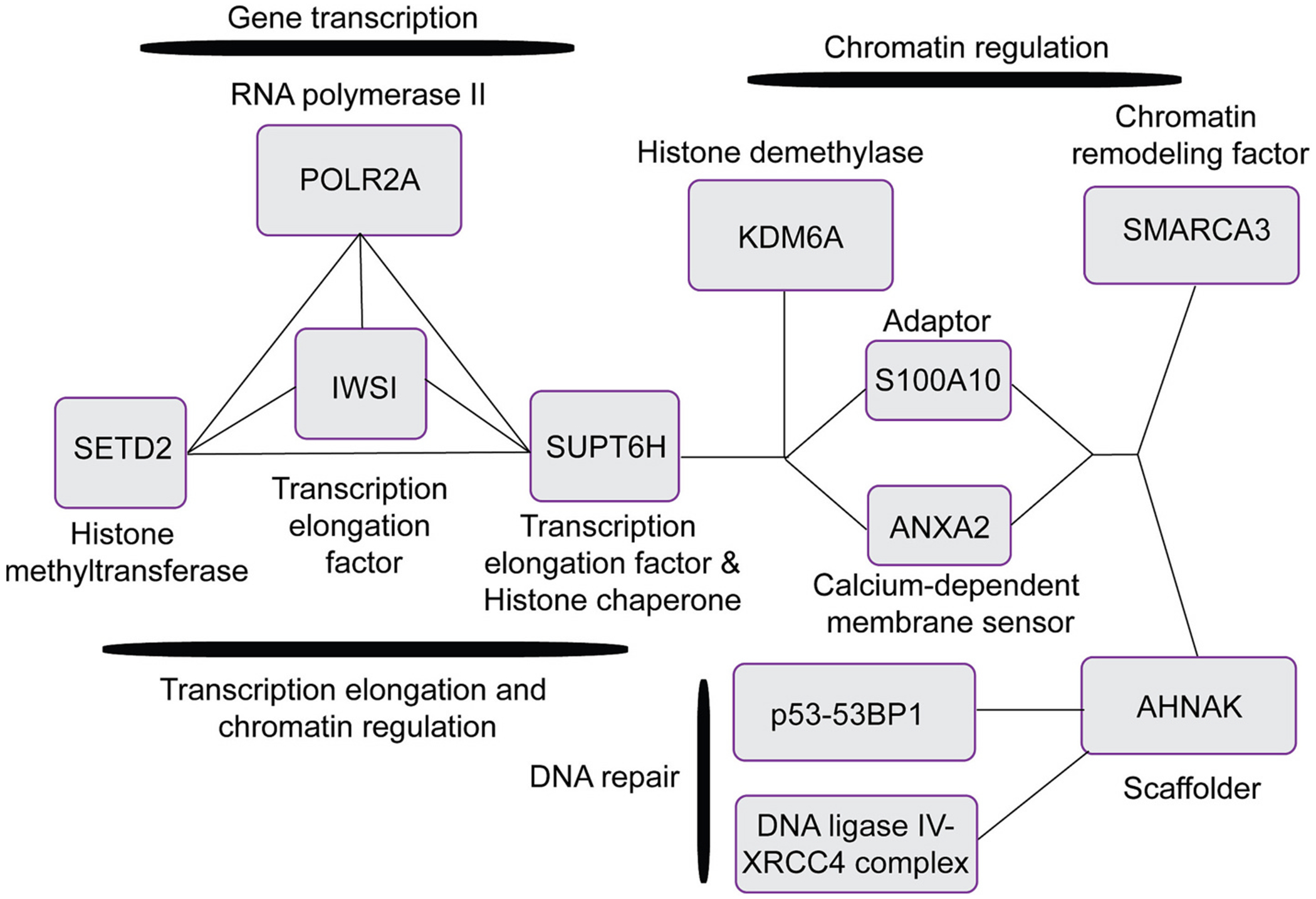
SUPT6H and its primary interaction partners. The connecting lines indicate functional links based on direct protein–protein interactions. The molecular functions and associated nuclear events mediated by each interaction partner are described.

In addition to the predicted deleterious effects of the 18 variants of *SUPT6H*, multiple lines of indirect evidence substantiate the involvement of SUPT6H in NDDs. SUPT6H directly interacts with the catalytic subunit of RNA polymerase II (PORLA2) [93,94], the transcription elongation factor IWS1 [95], and the mood disorder–associated proteins p11 (S100A10) and ANXA2 [31] (Fig. 7). SUPT6H also functionally interacts with the histone methyltransferase SETD2 [96] as well as histone demethylase KDM6A through direct interaction [97] or as a part of SUPT6H/S100A10/ANXA2 complex [85], contributing to the deposition of histone marks critical for chromatin organization and transcriptional fidelity [9]. SETD2 [16,98,99] and KDM6A [100,101] are established NDD genes. In addition, SUPT6H-mediated nuclear mechanisms might be affected by another chromatin-remodeling factor SMARCA3 and a scaffolding protein AHNAK *via* p11/ANXA2 complex [28,31] (Fig. 7). In the nucleus, AHNAK plays a role in DNA repair with 53BP1-p53 complex [102] and DNA ligase IV-XRCC4 complex [103]. The function of p11, ANXA2, SMARCA3 and AHNAK has been implicated in psychiatric disorders [28,31,53,54]. Given that proteins typically function through such interactions, these associations suggest that SUPT6H likely participates in shared molecular pathways underlying both neurodevelopmental and psychiatric conditions. Due to the widespread expression and ubiquitous role of SUPT6H in gene transcription, *SUPT6H* variants are likely to affect not only inhibitory neurons but also excitatory neurons, glial cells, and vascular cells in the brain. *Supt6* deletion in both excitatory and inhibitory neurons, or in excitatory neurons alone, is anticipated to cause more severe neuronal development defects and NDD pathogenesis. However, *Supt6* ablation in excitatory neurons might increase the likelihood of embryonic lethality. Collectively, findings from this study provide initial evidence suggesting that *SUPT6H* may be a potential neurodevelopmental gene.

## Supplementary Material

MMC2

MMC1

MMC4

MMC3

## Figures and Tables

**Table 1:** *SUPT6H* variants reported in individuals with developmental disorders and autism.

	cDNA change (NM_003170.5)	Amino acid change (NP_003161.2)	Exon number	Phenotype	gnomAD (allele frequency)	CADD 1.7 score	ACMG interpretation	Ref.
1	c.473C>T	p.Ala146Val (A146V)	5	Developmental disorder & autism	0.00000186	24.5	LP	[19,25]
2	c.523G>C	p.Asp175His (D175H)	5	Congenital heart disease	0	26	LP	[21]
3	c.767A>G	p.Lys256Arg (K256R)	7	Autism	0	22.9	LP	[22,24,25]
4	c.902G>A	p.Arg301His (R301H)	8	Developmental disorder & autism	0.00000434	29.7	LP	[19,25]
5	c.947A>C	p.Glu316Ala (E316A)	8	Congenital heart defects	0.00000867	27	LP	[23]
6	c.1138C>G^a^	p.Arg380Gly (R380G)	10	Neurodevelopmental disorder & autism	0	24.2	LP	[12,19,25]
7	c.1321C>T	p.Arg441Trp (R441W)	11	Developmental disorder & autism	0.00000248	33	LP	[19,25]
8	c.1471C>T	p.Arg491Cys (R491C)	12	Congenital heart defects	0.0000118	32	LP	[23]
9	c.1510G>A	p.Glu504Lys (E504K)	13	Developmental disorder & autism	0.0000211	23.4	LP	[19,25]
10	c.1549C>A	p.Gln517Lys (Q517K)	13	Autism spectrum disorder	0	21.7	LP	[22]
11	c.1559G>A	p.Arg520His (R520H)	13	Orofacial clefting	0.00000186	28.2	LP	[20]
12	c.2638A>G	p.Ile880Val (I880V)	21	Neurodevelopmental disorder & autism	0.0000124	18.65	LP	[12,19,25]
13	c.2924G>A	p.Arg975His (R975H)	23	Autism	0.0000223	32	LP	[25]
14	c.3194G>A^a^	p.Trp1065Ter (W1065*)	25	Developmental disorder & autism	0	39	P	[19,25]
15	c.4123G>T	p.Asp1375Tyr (D1375Y)	30	Autism spectrum disorder	0.0000273	32	LP	[22,25]
16	c.4484G>A^a^	p.Arg1495Gln (R1495Q)	33	Developmental disorder & autism	0.00000372	32	LP	[19,25]
17	c.4510G>A	p.Val1504 Met (V1504M)	33	Developmental disorder & autism	0.00000248	28.8	LP	[19,25]
18	c.4978C>T^a^	p.Arg1660Trp (R1660W)	36	Developmental disorder & autism	0.0000211	31	LP	[19,25]

a Indicates cases reported in Decipher (https://www.deciphergenomics.org/). P denotes pathogenic variants, and LP refers to likely pathogenic variants.

## Data Availability

Data will be made available on request.

## Appendix A. Supplementary data

Supplementary data to this article can be found online at https://doi.org/10.1016/j.bbadis.2026.168226.
