# Supplementary figures and images for: Genetic alterations in SUPT6H are associated with neurodevelopmental disorders
Source: Biochim Biophys Acta Mol Basis Dis. Author manuscript; Available in PMC 2026 Jun 12. (PMC13262130; doi:10.1016/j.bbadis.2026.168226)

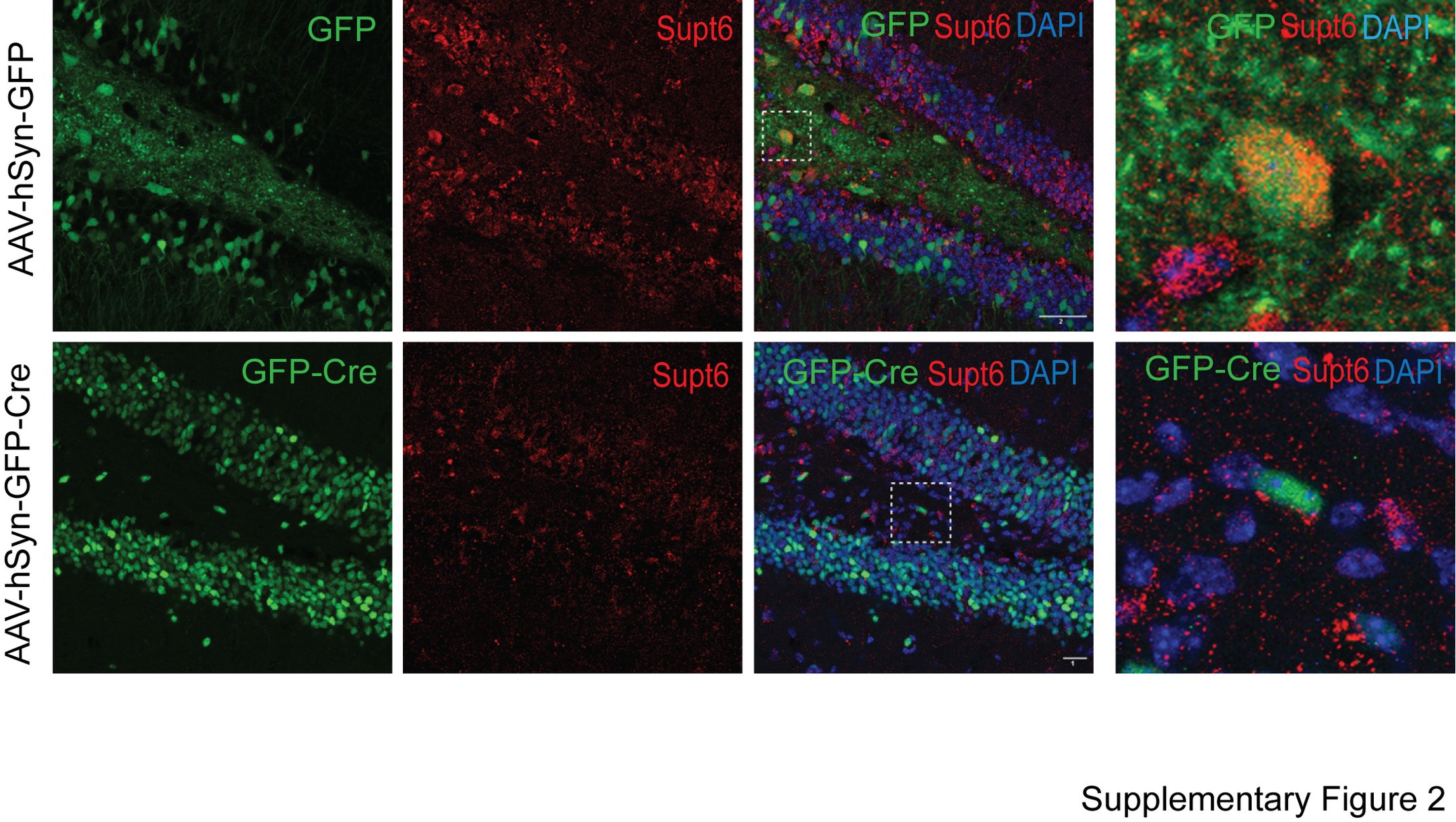

Supplement: MMC2 [file NIHMS2179584-supplement-MMC2.jpg]

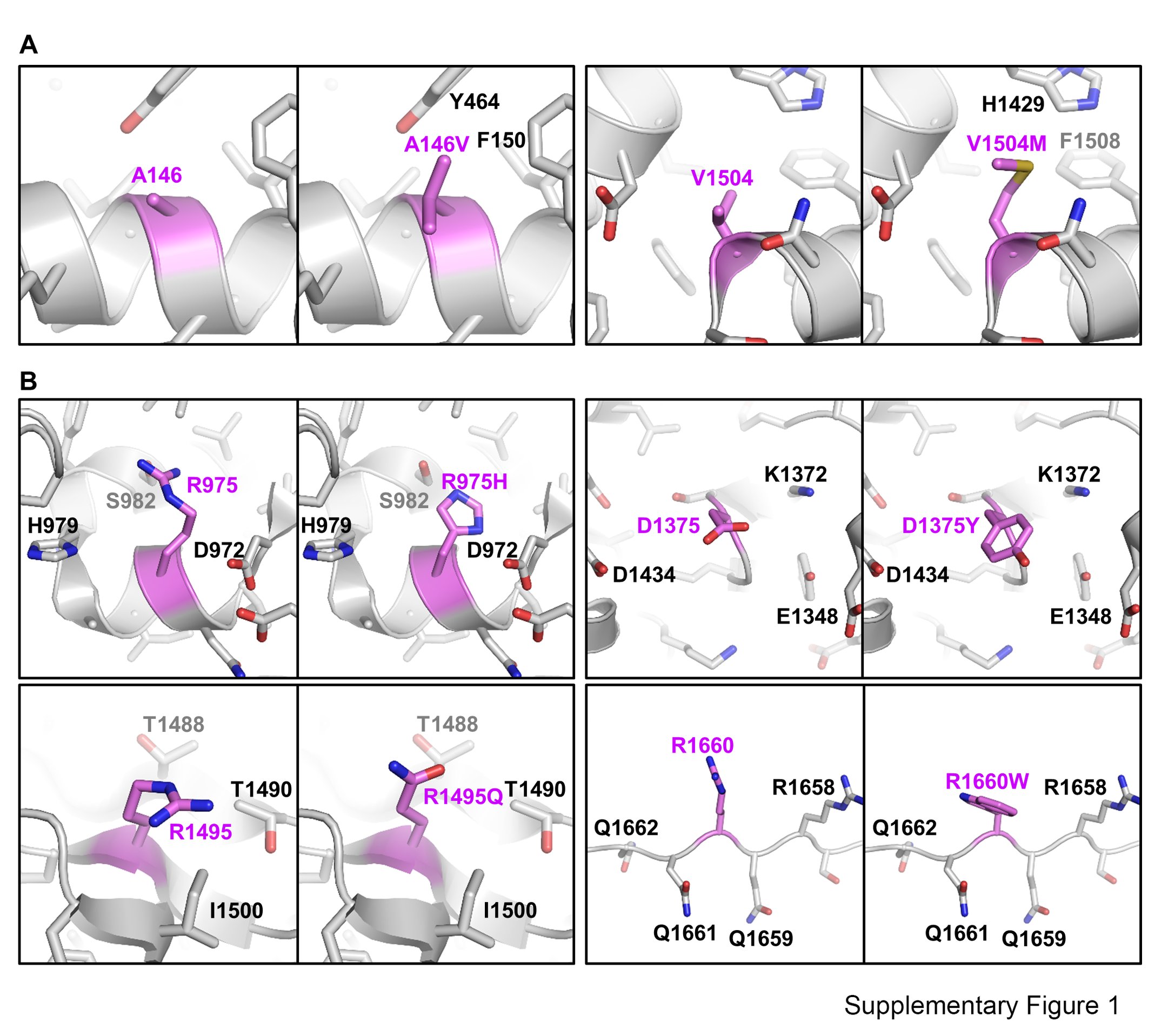

Supplement: MMC1 [file NIHMS2179584-supplement-MMC1.jpg]
